# Results-based financing to increase uptake of skilled delivery services in The Gambia: using the ‘three delays’ model to interpret midline evaluation findings

**DOI:** 10.1186/s12884-020-03387-9

**Published:** 2020-11-23

**Authors:** Laura Ferguson, Rifat Hasan, Chantelle Boudreaux, Hannah Thomas, Mariama Jallow, Günther Fink, The Project Implementation Committee (PIC), Modou Cheyassin Phall, Abdou A. Ceesay, Ousman Ceesay, Famata Colley, Modou L. Darboe, Malang N. Fofana, Catherine Gibba, Bakary Jallow, Musa Loum, Elizabeth Mago, Lamin Njie, Matty Njie, Alhagie Sankareh

**Affiliations:** 1 Institute on Inequalities in Global Health, University of Southern California, 2001 N Soto St, SSB318H, MC-9239, Los Angeles, CA 90089 USA; 2 World Bank Group, 70, Lodhi Estate, New Delhi, 110003 India; 3 Harvard Medical School, 641 Huntington Ave, Boston, MA 02115 USA; 4 Centre for Reproductive Sexual Health, Tanji, Kombo South, West Coast Region The Gambia; 5 Swiss Tropical and Public Health Institute, Socinstrasse 57, CH-4051 Basel, Switzerland; 6 National Nutrition Agency and Ministry of Health and Social Welfare, Birtil Harding Highway, Bakau, PMB 162, Banjul, The Gambia

**Keywords:** Maternity care, Results-based financing, The Gambia, Maternal health

## Abstract

**Background:**

Delays in accessing skilled delivery services are a major contributor to high maternal mortality in resource-limited settings. In 2015, the government of The Gambia initiated a results-based financing intervention that sought to increase uptake of skilled delivery. We performed a midline evaluation to determine the impact of the intervention and explore causes of delays.

**Methods:**

A mixed methods design was used to measure changes in uptake of skilled delivery and explore underlying reasons, with communities randomly assigned to four arms: (1) community-based intervention, (2) facility-based intervention, (3) community- and facility-based intervention, and (4) control. We obtained quantitative data from household surveys conducted at baseline (*n* = 1423) and midline (*n* = 1573). Qualitative data came from semi-structured interviews (baseline *n* = 20; midline n = 20) and focus group discussions (baseline *n* = 27; midline *n* = 39) with a range of stakeholders. Multivariable linear regression models were estimated using pooled data from baseline and midline. Qualitative data were recorded, transcribed, translated and thematically analyzed.

**Results:**

No increase was found in uptake of skilled delivery services between baseline and midline. However, relative to the control group, significant increases in referral to health facilities for delivery were found in areas receiving the community-based intervention (beta = 0.078, *p* < 0.10) and areas receiving both the community-based and facility-based interventions (beta = 0.198, *p* < 0.05). There was also an increase in accompaniment to health facilities for delivery in areas receiving only community-based interventions (beta = 0.095, p < 0.05). Transportation to health facilities for delivery increased in areas with both interventions (beta = 0.102, p < 0.05). Qualitative data indicate that delays in the decision to seek institutional delivery usually occurred when women had limited knowledge of delivery indications. Delays in reaching a health facility typically occurred due to transportation-related challenges. Although health workers noted shortages in supplies and equipment, women reported being supported by staff and experiencing minimal delays in receiving skilled delivery care once at the facility.

**Conclusions:**

Focusing efforts on informing the decision to seek care and overcoming transportation barriers can reduce delays in care-seeking among pregnant women and facilitate efforts to increase uptake of skilled delivery services through results-based financing mechanisms.

## Background

In 2015, an estimated 303,000 women died globally during pregnancy, childbirth or within 42 days of delivery [1]. Most of these deaths occurred in resource-limited settings and would have been preventable had appropriate services been accessed [2]. Delays in accessing skilled delivery services, therefore, are an important contributing factor to maternal deaths. The “Three Delays” model developed by Thadeus and Maine (1994) categorized these delays as follows: (1) delays in the decision to seek care, (2) delays in reaching an adequate health facility once the decision to seek care has been made, and (3) delays in receiving adequate care after arrival at the health facility [2].

Some results-based financing (RBF) programs have sought to increase uptake of timely institutional delivery but found no change in institutional delivery rates even when incentivizing this change [3]. However, in recent years, several RBF programs have been shown to increase rates of institutional delivery in resource-limited settings [4–9]. In most cases, this also implies increases in rates of skilled delivery, as skilled delivery, as defined by the World Health Organisation, is not available outside health facilities [10]. However, these studies also show the continued existence of all “Three Delays”. Delays in the decision to seek care can occur when family members oppose facility delivery, women fear facility delivery, women feel embarrassed by physical examinations at the health facility, family members feel no perceived need for facility delivery, women lack knowledge about the importance of facility delivery, or families lack the resources to pay for a facility delivery [4, 11–15]. Delays in reaching an adequate health facility can occur when women try to access a health facility from remote areas, women lack access to all-weather roads, labor begins at night or during poor weather, women lack someone to accompany them to a health facility, or women lack transportation or the means to pay for it [11–15]. Delays in receiving adequate care at the health facility can occur when health facilities lack the necessary resources and personnel for delivery or do not prepare for delivery in advance [11, 16].

Where the impact of RBF programs on increased uptake of institutional delivery has been well documented, there is little exploration of the mechanisms underlying changes in these trends. This study seeks to expand the knowledge base about factors contributing to the success of RBF programs by using the Three Delays model to frame an analysis of midline evaluation data from an RBF intervention in The Gambia. The intervention addressed a number of objectives relating to maternal and child nutrition and health, one of which was to increase uptake of skilled delivery. Here we report quantitative findings regarding the impact of incentivising activities related to skilled delivery uptake along with qualitative findings regarding the causes of delays in accessing skilled delivery. Quantitative data on referral to delivery are presented to help understand Delay 1; for Delay 2, quantitative data on accompaniment and transport to facility for delivery, facility delivery and delivery attended by a traditional birth attendant (TBA) are presented; for Delay 3, quantitative data on skilled delivery at the health facility are used. Qualitative data are then presented to further explore each delay and any changes between the two rounds of evaluation.

## Methods

### Study setting

The Gambia is one of the smallest countries in West Africa, with a population of approximately 1.9 million in 2013 [17]. The maternal mortality ratio and neonatal mortality rate are both high at 433 deaths/100,000 live births and 22 deaths/1000 live births respectively [18]. Maternal deaths accounted for 36% of all deaths among women aged 15–49 in 2013 [18]. Less than 60% of births were attended by skilled providers, and only 63% of births occurred in health facilities [18].

The overarching goal of the national health policy is to reduce morbidity and mortality through the provision of equitable, affordable and quality health services and related services [19]. Regarding skilled delivery, this encompasses ensuring the availability of skilled attendants for the provision of basic essential obstetric and newborn care within a “functioning healthcare setting” as well as 24-h access to comprehensive emergency obstetric and newborn care [20].

Historically, TBAs have played an important role in assisting women during pregnancy, delivery and the post-partum period. In January 2015, the Ministry of Health and Social Welfare redesignated TBAs as “community birth companions” (CBCs) and redefined their role. This change was based on the recognition that despite the importance of their support to women, the inability of TBAs to provide emergency obstetric care has hampered efforts to reduce maternal mortality. Their new role was envisaged as referring and/or escorting women during labor or childbirth, with or without complications, to health facilities within their catchment areas and providing antenatal and postnatal care [19]. With this shift in policy, the government advised that all deliveries should take place within health facilities and be attended by skilled personnel.

### Intervention

The Maternal and Child Nutrition and Health Results Project (MCNHRP), supported by the World Bank and the Health Results Innovations Trust Fund, was an initiative undertaken by the government of The Gambia to improve maternal and child health outcomes, with one objective being to increase uptake of skilled delivery. It was designed to be implemented in the three regions of The Gambia with the poorest health indicators: the Upper River, the Central River and the North Bank West Regions (URR, CRR and NBR-W). One-third of the total population of The Gambia lives in these regions.

Preliminary assessments conducted to guide the design of the MCNHRP identified both supply-side and demand-side obstacles to achieving the desired health outcomes, and suggested that interventions should be purposefully integrated into existing community structures and the government health system. Taking into account these considerations, it was determined that the MCNHRP would incorporate RBF mechanisms into two core intervention packages: one implemented at the health facility level, and the other implemented at the community level.

The facility-based intervention package was largely intended to address supply-side barriers identified in the preliminary assessments. It incentivized the provision of specified maternal and child nutrition and health services, including facility-based delivery by a skilled practitioner, as well as incentivizing service quality. It was implemented at hospitals, major health centers and minor health centers. Quarterly payments were issued to facilities on a fee-for-service basis. The incentivized service that we report on in this study, skilled delivery at a health facility, was rewarded with a payment of approximately 525 Gambian dalasis (US$ 12.50) per skilled delivery performed. The facilities could earn an additional amount up to the equivalent of 100% of the quarterly payment for full compliance with quality standards. The quality assessment tool addressed a range of issues such as cleanliness, quality of record-keeping, and availability of staff and supplies. Each health facility could use the RBF payments that it received to finance items in its quarterly business plan, such as materials and equipment, drugs, training, consulting services, and other operating costs. A maximum of 40% of payments could be allocated to staff bonuses. As a part of the facility-based intervention, women were invited to enroll in a conditional cash transfer (CCT) program in which they received one payment for attending an initial ANC visit in the first 12 weeks of pregnancy and a second equivalent payment for completing at least three more ANC visits during the course of the pregnancy.

The community-based intervention package sought to encourage community mobilization activities and social and behavioral change communication (SBCC) to overcome demand-side barriers identified in the preliminary assessments. It provided quarterly incentive payments to village development committees (VDCs) and village support groups (VSGs) for achieving specified community targets relating to maternal and child health and nutrition, including a target for the number of pregnant women referred to health facilities for delivery. VDCs serve as the lowest local level of governance in The Gambia, and VSGs conduct health and nutrition education and mobilization activities. A VSG is comprised of three men and five women, including a village health worker and community birth companion, all of whom carry out their duties on an unpaid basis. The overall purpose of the VSGs is to increase knowledge and awareness of maternal and child health and nutrition, promote the adoption of healthy behaviours and good nutrition practices, and encourage appropriate care-seeking. In communities that received the community-based intervention package, VSGs were asked to carry out SBCC activities intended to increase knowledge and awareness of pregnancy, labor and delivery, as well as to promote institutional delivery.

Eighty percent of each incentive payment that a community earned was allocated to the VDC for implementing community development activities elaborated in a quarterly business plan. The VSG received the remaining 20% for distribution among its members as incentive payments for conducting SBCC activities.

In the three regions of The Gambia where MCNHRP implementation took place, there are 22 health centers and two hospitals serving a total of approximately 800 communities. The interventions were first pilot tested in three health centers and their catchment areas in North Bank West Region. Following pilot testing, the interventions were then refined for implementation in the remaining 19 health centers and their catchment areas, with rollout staggered to allow for comparisons between communities reached at different times. Facility- and community-based interventions were independently randomized preceding implementation. Lots were drawn in a public ceremony to select the ten health facility catchment areas that would receive the facility-based intervention in the first phase of the study. Participation in the community-based intervention was similarly determined with the random selection of communities from each health facility catchment area.

The National Nutrition Agency and Ministry of Health and Social Welfare jointly implemented the interventions in collaboration with regional health directorates, health facilities and communities.

### Evaluation design

We used a randomised 2 × 2 study design to measure the community-level impact of three intervention arms compared with the control arm: the facility-based arm, the community-based arm, and the combination facility-based and community-based arm. (Table 1).

**Table 1:** Study design

		Facility-based intervention	
		Comparison	Intervention
**Community-based intervention**	Comparison	*No intervention*	*Facility intervention only*
	Intervention	*Community intervention only*	*Facility + community interventions*

Prior to the launch of MCNHRP activities, it was determined that two-stage cluster sampling would be carried out to obtain evaluation data. Accordingly, in the first sampling stage, six communities from the catchment area of each of the 19 non-pilot health facilities were randomly selected to participate in the evaluation, with the most recent census estimates used as the basis for probability proportional to population size sampling. These communities would be visited in each round of data collection. Due to a technical failure with the tablets used to collect information, data from one community were lost at baseline. Thus, the final sample included 112 communities at baseline and 113 at midline. Baseline data were collected in October–November 2014, shortly before the launch of program activities. Midline data were collected approximately halfway through the overall study period, in July–August 2016.

At the time of midline data collection, the facility-based component of MCNHRP had been implemented at 10 non-pilot health facilities for approximately 18 months. Their catchment areas collectively encompassed 60 evaluation communities (Table 2). Additionally, the community-based component of MCNHRP had been implemented in 37 evaluation communities across the 19 health facility catchment areas for approximately 15 months. Twenty of the 37 evaluation communities were in catchment areas where the facility-based intervention also was taking place, and the remaining 17 were in catchment areas that did not receive the facility-based intervention. The 36 evaluation communities that were not exposed to either intervention served as controls.

**Table 2:** Distribution of evaluation communities among study arms

	Number of Health Facilities	Number of Communities
Facility Yes; Community Yes	10	20
Facility Yes; Community No	10	40
Facility No, Community Yes	9	17
Facility No, Community No	9	36

A qualitative study was embedded within the quantitative study to allow for triangulation across data sources.

### Quantitative data collection

All quantitative data used in this analysis were drawn from household surveys administered at baseline and midline to heads of households and mothers of children under the age of five. Survey instruments were adapted from the household survey in the World Bank’s impact evaluation toolkit for results-based financing [21]. Experienced enumerators from the Gambia Bureau of Statistics were trained on and then administered these surveys with technical support from the evaluation team. The surveys collected information on household demographics, socioeconomic variables, healthcare utilization and health outcomes. All quantitative survey data were collected on tablets with real-time data quality checks.

A target of 2400 households was chosen in order to be able to detect an improvement of 10 percentage points in the main outcome variables with power 0.9 and an intra-class correlation coefficient of 0.05. For skilled deliveries at facility, baseline prevalence in the randomized areas (19 non-pilot health facilities) was 0.42. The study was powered to detect a 16 percentage point increase with power 0.8, and an 18 percentage point increase with power 0.9. Despite the use of baseline data, the ex-post design effect was large (DEFF = 4.1) for the facility-based intervention due to the small number of clusters (*N* = 19) in the study, somewhat limiting the ability to detect project impact. Community randomization was done at the cluster level. For variables with a baseline prevalence of 50% (such as institutional deliveries), the study was powered to detect a 10 percentage point increase with power 0.8, and a 12 percentage point increase with power 0.9.

For each round of the household survey, two-stage cluster sampling was used to identify a random sample targeting 120 households from each of the 19 health facility catchment areas included in the evaluation. As explained previously, evaluation communities were selected in the first sampling stage. Immediately prior to each round of survey activities, each evaluation community was visited by mapping teams to develop a list of eligible households. From all eligible households listed, 20 households were randomly selected for the survey. If fewer than 20 households in a given community were eligible, all would be selected. A household was eligible for inclusion if it had at least one woman aged 15 or older and at least one child under the age of five. A mother was eligible to participate if she was at least 15 years of age and her child was under five years of age. Within each household, questionnaires were administered to the head of the household and the mother of the youngest child. The mother was asked to respond to questions about delivery practices in relation to her most recent delivery.

### Quantitative data analysis

This analysis was restricted to households with a birth within the 450 days (approximately 15 months) preceding the survey (whether baseline or midline), which approximately restricts the midline analysis to women who gave birth since the program “fully” launched. It utilises six indicators to assess whether the intervention benefitted maternal and child health by encouraging the uptake of health services and behaviors: referred to delivery, accompanied to delivery, transported to delivery, community birth companion, facility delivery, and skilled delivery at facility, each of which is described below:

1. *Referred to delivery.* Pregnant woman was referred by a community birth companion to a health facility for delivery.
2. *Accompanied to delivery.* Pregnant woman was accompanied by a community birth companion to a health facility for delivery.
3. *Transported to delivery.* Pregnant woman was provided with transportation to a health facility for delivery.
4. *Community birth companion.* Pregnant woman delivered at home with a community birth companion. (In the context of The Gambia, a community birth companion is an unskilled health provider.)
5. *Facility delivery*. Pregnant woman delivered at a health facility (minor health center, major health center, or hospital).
6. *Skilled delivery at facility.* Pregnant woman delivered at a health facility, attended by a skilled health provider.

Two indicators, ‘referred to delivery’ and ‘skilled delivery at facility’, were directly incentivized by the MCNHRP interventions.

Multivariable linear regression models for all six indicators were estimated using pooled baseline and midline data. Regression models included indicator variables for midline survey (time trend) and each of the three intervention arms at the time of data collection (facility-based intervention only, community-based intervention only, and facility-based intervention plus community-based intervention). To account for spatial differences, settlement fixed effects were also included in all models. Standard errors were clustered at the facility level using Huber’s cluster robust variance estimator [22]. Beta coefficients and standard errors are presented to show intervention impact across different study arms.

### Qualitative data collection

Based on an embedded mixed methods design, primary qualitative data were collected from focus group discussions (FGDs) and key informant interviews to help gain nuanced insight into people’s experiences of the project as well as the reasons underlying the project’s performance (Table 3). Study communities were purposively selected to reflect a mix of levels of performance across quantitative indicators as well as regional diversity. Different guides were used for each type of participant but core themes explored included: understanding of the MCNHRP, perceptions of the project, health and nutrition-related behaviors within the community, and how these behaviors may have changed since project inception.

**Table 3:** Qualitative participants and sample sizes

Participants	Sample Size	
	Baseline (Jan-Feb 2015)	Midline (July – August 2016)
**Focus group discussion**	**# of FGDs (# of participants)**	
RBF Committee	1 (6)	1 (6)
Project Implementation Committee	1 (6)	1 (8)
Regional Health Directorate (RHD)	3 (18)	3 (17)
Health workers	3 (17)	5 (22)
Catchment Area Committee	3 (18)	3 (16)
VDC/VSG	5 (32)	7 (45)
Women who had delivered in the previous six months	6 (51)	13 (74)
Men	5 (35)	6 (35)
*Total focus group discussions*	*27 (193)*	*39 (223)*
**Key informant interview**	**# of interviews**	
Health Facility Officer-in-Charge	3	3
Community Health Nurse	3	3
TBA/CBC	5	7
Vulnerable women^a^	9	7
*Total interviews*	*20*	*20*

^a^‘Vulnerable’ was not specifically defined but was determined by community leaders during data collection. Typically it included widows and unmarried adolescents

FGDs lasted from 90 to 150 min, while interviews lasted from 45 to 90 min. Nobody refused participation or dropped out partway through a FGD or an interview.

Three local qualitative researchers – one nurse as team lead (female, currently working for a sexual and reproductive health community-based organisation) and two community health nurses (one female, currently working at the national family planning association; one male, currently working as a monitoring and evaluation officer for an NGO) – collected the qualitative data in the language of the respondents’ choice. All team members had prior experience working in the field of maternal and child nutrition and health, and carrying out interviews and/or FGDs. Prior to each round of data collection, the team was trained on the interview and FGD guides. Instruments were field tested and amended as necessary before data collection began.

There was no prior contact between the researchers and study participants. Participants were purposively sampled and recruited through face-to-face contact with community gate-keepers. Researchers introduced themselves to participants, explained the purpose of the study, answered any questions and sought informed consent before data collection. Participants helped to decide the most appropriate location for each interview/FGD to ensure a ‘safe’ space without the presence of anyone besides the participants and researchers. Researchers took field notes throughout data collection. The same process was used at baseline and midline with no attempt to find the same participants at midline as had been included at baseline.

### Qualitative data analysis

All interviews and focus group discussions were recorded, transcribed verbatim and translated to English. The transcripts, along with the researchers’ field notes, were entered and analyzed in NVivo 10. Data were double-coded and thematically analyzed using a framework derived originally from the literature and then refined as themes emerged in the data. The same code tree was used for the baseline and midline analyses (available on request). Quotes that are illustrative of the main themes that emerged are provided.

### Joint data analysis

Once independent analysis of the quantitative and qualitative data was complete, data were triangulated, which we understood to mean “a process of studying a problem using different methods to gain a more complete picture”. Areas of convergence, complementarity and divergence were identified, and the explanatory value the qualitative data could afford quantitative findings explored [23].

### Ethical approval

Ethical approval for the impact evaluation was obtained from The Gambia Government/MRC Joint Ethics Committee (R014036v2) as well as the Ethics Review Committee of the University of Southern California (HS-14-00688). Study participation was voluntary, and informed consent was obtained from all study participants after they were told about the study objectives and about how their information would be used.

## Results

### Survey administration

Survey participation rates were high. After the ex-post data restriction to include only households with a woman with a birth in the last 450 days (15 months), there were 1490 households approached at baseline, all of which consented to participate. Of these, 1423 (96%) completed the survey. At midline, after the ex-post data restriction to include only households with a woman with a birth in the last 450 days, 1573 households were eligible, all of which consented and completed the survey. Improvements in technology between the baseline and midline survey rounds help to explain the reduction in incomplete interviews at midline.

### Household survey findings

Characteristics of mothers with a birth in the prior 450 days in each of the study arms and overall are reported in Table 4, which shows that there were very few significant differences between the baseline sample and the midline sample. Only the proportion of women who have never been married and the proportion of women of Wolof ethnicity are significantly higher in the midline sample than in the sample at baseline. The last six rows of the table show the mean values for each of the six primary outcomes of interest in this paper at baseline and midline. This can help contextualize the beta coefficients from the regression models that are presented below.

**Table 4:** Characteristics of mothers with a birth in the prior 450 days

	Round 1					Round 2					Test for Difference, Overall
	Control	Facility Only	Community Only	Facility and Community	Overall	Control	Facility Only	Community Only	Facility and Community	Overall	
***Demographic Data***											
Mean Maternal Age	27.5	27.3	27.0	26.7	**27.2**	27.2	27.7	26.8	27.0	**27.3**	0.842
Mother’s age: < 20	11.9%	11.1%	11.5%	11.8%	**11.5%**	11.8%	9.2%	10.8%	12.8%	**10.9%**	0.645
Mother’s age:20–34	71.6%	73.4%	73.8%	74.2%	**73.0%**	72.0%	73.2%	75.3%	72.8%	**73.1%**	0.972
Mother’s age: > 34	16.5%	15.6%	14.7%	14.0%	**15.5%**	16.2%	17.5%	13.9%	14.4%	**16.0%**	0.721
Monogamously married	56.2%	57.0%	61.3%	58.4%	**57.7%**	57.8%	56.4%	65.7%	54.4%	**57.9%**	0.946
Polygamously married	42.6%	41.2%	38.7%	41.0%	**41.2%**	40.5%	41.6%	33.7%	43.6%	**40.4%**	0.712
Never married	0.3%	0.5%	0.0%	0.6%	**0.4%**	0.6%	1.0%	0.6%	1.5%	**0.9%**	0.107
Widowed or divorced	0.9%	1.3%	0.0%	0.0%	**0.7%**	1.2%	1.0%	0.0%	0.5%	**0.8%**	0.806
CRR	42.3%	46.7%	44.0%	55.1%	**46.2%**	47.4%	47.0%	45.2%	55.9%	**48.4%**	0.300
NBR West	10.5%	8.8%	7.9%	7.9%	**9.0%**	10.1%	10.9%	11.4%	9.2%	**10.5%**	0.251
URR	47.2%	44.5%	48.2%	37.1%	**44.8%**	42.5%	42.1%	43.4%	34.9%	**41.1%**	0.083
Ethnicity: Mandinka/ Jahanka	23.0%	46.7%	23.0%	30.3%	**32.6%**	15.6%	45.3%	28.3%	33.8%	**31.6%**	0.597
Ethnicity: Fula/Tukolor	17.0%	14.8%	21.5%	19.7%	**17.4%**	16.2%	13.1%	15.7%	17.9%	**15.3%**	0.173
Ethnicity: Wolof	20.7%	9.8%	31.4%	24.7%	**19.3%**	28.9%	14.6%	34.9%	28.2%	**24.4%**	0.003
Ethnicity: Sarahule	35.8%	26.4%	20.4%	19.7%	**27.3%**	37.0%	22.9%	18.1%	17.4%	**25.6%**	0.369
Ethnicity: Other	3.4%	2.3%	3.7%	5.6%	**3.4%**	2.3%	4.1%	3.0%	2.6%	**3.1%**	0.724
Average Number of Children Ever Born	3.9	4.0	3.9	3.9	**3.9**	4.0	4.2	3.8	4.2	**4.1**	0.146
Average Number of Children Surviving	3.6	3.6	3.6	3.7	**3.6**	3.7	3.8	3.5	3.9	**3.8**	0.180
***Outcome Variables of Interest***											
Referred to Delivery	15.5%	14.4%	14.3%	14.1%	**14.7%**	24.2%	27.8%	31.5%	44.6%	**30.2%**	0.000
Accompanied to Delivery	12.9%	17.0%	14.3%	20.3%	**15.8%**	26.5%	32.5%	38.2%	48.2%	**34.2%**	0.000
Transported to delivery	4.3%	6.9%	6.3%	4.0%	**5.5%**	12.5%	11.1%	18.2%	23.8%	**14.8%**	0.000
Institutional Delivery	56.2%	57.3%	55.0%	66.7%	**58.1%**	55.7%	57.9%	64.2%	63.4%	**59.1%**	0.620
Skilled birth attendence	43.0%	47.0%	45.0%	48.0%	**45.6%**	61.7%	61.8%	66.1%	63.4%	**62.7%**	0.000
Community birth companion	43.8%	42.4%	43.4%	45.2%	**43.5%**	28.7%	31.0%	26.7%	27.8%	**29.1%**	0.000
Skilled delivery at facility	21.2%	20.1%	20.1%	15.8%	**19.7%**	51.0%	45.8%	54.5%	54.6%	**50.3%**	0.000
N	352	398	191	178	**1119**	346	411	166	195	**1118**	

**Fig. 1:**
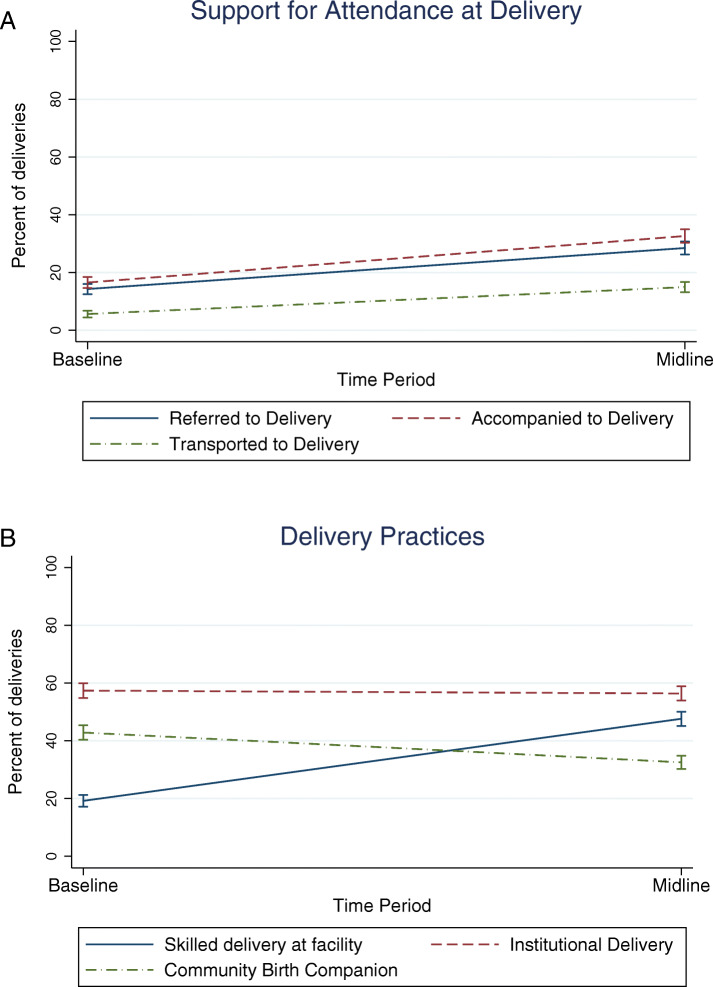
**a** Time trend for support for attendance at delivery. **b**. Time trend for delivery practices

The strong time trends for the outcome variables of interest seen in Table 4, were all statistically significant except for the change in institutional delivery. Two of these are illustrated in Fig. 1a and b.

The interventions were associated with statistically significant positive changes in some indicators related to the uptake of skilled delivery services, but not others. Significant increases in referral to health facilities for delivery were found in areas receiving the community-based intervention (beta = .078, *p*-value <.10) and areas receiving both the community-based and facility-based interventions (beta = .198, p-value <.05) (Table 5). There was also an increase in accompaniment to health facilities for delivery in areas receiving only the community-based intervention (beta = .095, *p* < .05). For transport to delivery, increases were found only in areas receiving both interventions (beta = 0.102, p-value > .05).

**Table 5:** Changes in referral, accompaniment and transportation to the health facility for delivery (*N* = 2211 observations)

Variables	Referred to delivery	Accompanied to delivery	Transported to delivery
Facility-based intervention only	0.0401	0.015	−0.0468
	(0.053)	(0.052)	(0.039)
Community-based intervention only	0.0783*	0.0947**	0.029
	(0.038)	(0.037)	(0.047)
Facility-based intervention plus community-based intervention	0.198**	0.114	0.102**
	(0.083)	(0.068)	(0.045)
Time trend (midline dummy)	0.0974***	0.144***	0.0894***
	(0.027)	(0.031)	(0.029)
Constant (mean at baseline in control area)	0.145***	0.158***	0.0545***
	(0.014)	(0.013)	(0.009)
Observations	2211	2211	2211
R-squared	0.152	0.161	0.13

Analysis controls for settlement fixed effects. Robust standard errors in parentheses

*** *p* < 0.01, ** *p* < 0.05, * *p* < 0.1

Although increases in intermediate indicators such as referral, accompaniment and transportation to the health facility for delivery can be expected to lead to an increase in institutional delivery, no significant impact was found on institutional delivery, skilled delivery or delivery by a community birth companion (Table 6). However, it is important to note that there was a significant negative time trend for births with a community birth companion and a significant positive time trend for skilled attendance within a facility; these are trends that reflect overall improvements in delivery practices. Thus, to attain statistical significance, the impact of the interventions above and beyond this would have needed to have been large enough to overcome these time trends.

**Table 6:** Changes in delivery practices (*N* = 2214 observations)

Variables	Community birth companion	Facility delivery	Skilled delivery at facility
Facility-based intervention only	0.0541	0.0025	−0.0496
	(0.073)	(0.055)	(0.083)
Community-based intervention only	0.0122	0.0644	0.00254
	(0.062)	(0.076)	(0.080)
Facility-based intervention plus community-based intervention	−0.00747	−0.0504	0.0686
	(0.092)	(0.086)	(0.094)
Time trend (midline dummy)	−0.163***	0.00987	0.322***
	(0.055)	(0.045)	(0.053)
Constant (mean at baseline in control area)	0.434***	0.580***	0.192***
	(0.018)	(0.013)	(0.020)
Observations	2214	2214	2214
R-squared	0.185	0.243	0.231

Analysis controls for settlement fixed effects. Robust standard errors in parentheses

*** *p* < 0.01, ** *p* < 0.05, * *p* < 0.1

In qualitative interviews, women and health workers reported large increases in the number of health facility deliveries being sought and performed over the 18 months prior to the midline evaluation. This corresponds to the quantitative findings regarding statistically significant increases in referral, accompaniment and transportation for delivery among women who were reporting on their most recent birth in the last five years. However, the qualitative finding regarding perceived increases in health facility deliveries is at odds with the finding for the related quantitative indicator.

#### Understanding factors affecting each ‘delay’

Qualitative data show the continued existence of delays according to the “Three Delays” model. Delays 1 and 2 persisted while delay 3 was not often mentioned as much of a challenge as women received care quickly upon reaching the health facility. At the baseline and midline evaluations, women, health workers and VSG members described many of the same underlying causes of delays. However, in many cases fewer respondents described each cause of delay at midline compared to at baseline (Table 7).

**Table 7:** Factors Key Informants Discussed to Explain Delays

	***Baseline Only***	***Baseline and Midline***	***Midline Only***
**Delay 1**	• Concealment of labor	• Preference for home delivery • Limited knowledge of delivery indications • Delay in informing others after labor onset	• Reluctance to be delivered by a male midwife
**Delay 2**		• Lengthy care seeking process^a^ • Commencement of labor at night or after rain • Lack of effective available transportation • Cost of transportation • Long transport times	
**Delay 3**	• Inattentive staff	• Drug stock outs	• Minimal delays • Improved quality of care

^a^Before transportation to the health facility, women traditionally inform several family members, receive an examination from the Community Birth Companion, and either they or their husbands spend time searching for transportation

In 2014, delays in the decision to seek care usually occurred when women preferred home delivery, or had limited knowledge of delivery indications. In some cases, home delivery occurred when women concealed their labor. One CBC explained: “*[some women] hide their labor from the people around them until the baby is born then they send to call me to come.”* [Interview, CBC, NBR-W]. Delay in deciding to access care resulting from limited knowledge of delivery indications occurred frequently, primarily when women misunderstood their due dates or misidentified labor pains. In addition, some women delayed calling the CBC so that she only arrived at the time of delivery to avoid the pain and discomfort of being transported to the hospital on a donkey cart while in labor. This mode of transportation was not acceptable to them.

In 2016, no woman described concealment of labor at the midline evaluation. However, delays due to limited knowledge of delivery indications and preference for home delivery persisted. Although some women still lacked knowledge of delivery indications, women were more commonly able to describe their expected due dates and identify labor pains. Reports persisted of women who did not immediately seek care when labor began. One woman described, “*When I feel the pain, it was not so much so I waited until the pain was more then I told my mother and she went to call [the Community Birth Companion]*.” [Interview, Divorced woman, NBR-W]. A minority of women expressed a preference for home delivery, usually because they were used to home delivery or were afraid of the health facility. Additionally, some women, particularly in CRR, decided not to seek skilled delivery because of a reluctance to be delivered by a male midwife. One health worker explained: “*There are some women here, they would rather die at home than come here during childbirth because there is no female midwife*.” [FGD, Health workers, CRR] Geography was not cited as a factor in the preference for home delivery.

Once they have decided to seek care and before going to the health facility, women traditionally inform several family members, and receive an examination from the CBC before trying to find a means of transportation to attend the health facility. Even after consulting family members and the CBC, it can take a long time to locate a mode of transportation for reaching the health facility. Women who preferred home delivery most commonly cited the distance to the health facility as the reason for their preference. In 2014, many women described delays in reaching a health facility due to unavailability of effective transportation. One woman described, *“If your husband does not have a horse cart or cannot hire one, you have to deliver in the village, even if you want to deliver at the health facility. Mobility is the main cause of delivery in the village.”* [FGD, Women who had delivered in the previous 6 months, NBR-W]. Communities expressed that having access to horse carts, vehicles, or motorcycles was useful in reducing this type of delay. However, several limitations of animal-drawn transportation were described in addition to the persistent reports of discomfort during labor. This method was often too slow or unsuitable to the terrain. For example, in two communities, the health facilities were located on top of hills. Because donkey carts were unable to haul heavy loads uphill, delays resulted when pregnant women had to climb by foot to reach the health facility. Delay 2 also occurred due to long transport times, costs associated with transportation, and a lengthy care seeking process. Delay 2 was more common if labor commenced at night, during and just after rains. Reflecting the quantitative findings, women and community birth companions alike described newfound emphasis on referral, and accompaniment to delivery.

In 2016, delays due to the lengthy care-seeking process, long transport times, and the cost of transportation persisted. Although a lack of effective available transportation continued to contribute to Delay 2 in some places, some communities reported having acquired vehicles for transportation (often using RBF subsidies to finance these vehicles). Communities indicated that procurement of a vehicle for this purpose was one of the most beneficial aspects of the project. Transportation difficulties were again more frequent at night, during and after rains.

Delay 3 was described infrequently in 2014. Though most women expressed that they were adequately treated at the health facility, some community members described delays resulting from inattentive staff. One CRR man described, *“The way I was received when I escorted my wife who was in labor was not the least satisfactory to me. When I arrived ... the staff said they were having their breakfast and didn’t have time for me … When my wife called for assistance I found her in full labor and she delivered in my hands.” *[FGD, VDC & VSG members, CRR].

Delay 3 was reported minimally in 2016. Across all three regions, women reported being cared for quickly, adequately supported by staff, and provided with appropriate amenities and treatment. *“[In the past] you would sit there suffering but since this RBF project came they immediately receive, take you inside and give you the care. In the past a woman could even give birth in the open but not anymore.”* [FGD, Women who had delivered in the previous 6 months, NBR-W] In some regions, particularly in areas receiving facility and community interventions, the interviews with health facility staff suggest great improvements, but some community members still feel as though attitudes could be better and wait times shorter. There were also still a few reports of disrespect by health staff, and in some cases being shouted at by nurses.

Far more than occurred at baseline, many women described receiving quality services at the time of delivery, including being treated kindly by health workers: *“The other improved area that they mentioned is the attitude of the health service providers which is in its highest positive level now compared to before the project started. They talked about the lack of patience and poor attitude shown to them by the health staff in the past. This even made some women and men not wanting to seek health care from the said health facility. But this is all history now because of the project, they said. There is a good relationship between the staff and the health service users now to the level of building personal friendship with the health staff.”* [Interviewer notes, FGD, Women who had delivered in the previous 6 months]. In addition, administrative data show that a higher proportion of facility deliveries were performed by skilled health personnel at midline compared to at baseline, which also is indicative of better quality of care with respect to clinical processes and an improvement in Delay 3.

However, health workers reported some concerns that insufficient supplies continue to detract from the quality of delivery services. One NBR-W health facility employee described, *“It is not easy to have all [women deliver here], as at the labor ward we don’t have enough delivery beds. There is only one delivery bed.”* [FGD, Health workers, NBR-W].

Although not mentioned specifically in the context of delivery services, stockouts of essential medicines were almost ubiquitous at baseline. In 2016, people reported that the situation had improved, in part because health facilities were using part of their incentive payments to purchase drugs, but stockouts persisted at some facilities which might have impacted the ability to provide quality delivery services.

Based on qualitative data, Delays 1 and 2 persisted between 2014 and 2016, though in many cases fewer reports of delay were expressed in 2016. Delay 3 seems to have contributed the least to overall delays, with a negligible contribution by 2016.

## Discussion

This rigorous, randomized study with an embedded qualitative component is, to our knowledge, the first mixed methods assessment of an RBF intervention that comprises both a facility and community intervention. It aimed to assess the impact of these RBF interventions on a range of maternal and child health and nutrition outcomes, including the uptake of skilled delivery services at health facilities. The data presented here constitute compelling evidence of significant change across some, but not all, indicators of interest as well as some insights into the experience of care from provider and client perspectives. Delays in deciding to seek care and in reaching health facilities might continue to constitute barriers to the uptake of institutional and skilled delivery. If intermediate indicators/precursors like referral, accompaniment and transportation to health facilities for delivery continue to increase, facility deliveries and skilled deliveries within facilities should increase concomitantly with continued exposure to the interventions.

### Contextualizing findings on the three delays

The increase in the proportion of women who reported having been referred to health facilities for delivery may be linked to reducing ‘Delay 1’ around the decision to seek care at the time of delivery. Qualitative data reveal some factors contributing to Delay 1 that have not been previously explored. At this study’s baseline evaluation, women expressed that lack of knowledge of delivery indications and the concealment of labor contributed to Delay 1. This may occur because, in The Gambia, women are brought up to believe that they should bear pain quietly as a sign of strength, including labor pain. However, 18 months later no women described concealing their labor. During this period, intensive SBCC activities on the benefits of early ANC registration and facility delivery were implemented, which may explain the apparent reduction in the number of women concealing their labor. Understanding cultural approaches to bearing pain and addressing these through SBCC efforts can help promote timely uptake of skilled delivery in The Gambia and elsewhere.

Many of the underlying causes of delay noted by this study are confirmed by existing literature [11, 13, 24–26]. For example, women’s desire to consult with multiple family members before seeking delivery care, which caused delays was also identified among women in a study of RBF communities in India [13].

The finding that some women may be reluctant to seek skilled delivery where midwives are male has also been seen in Ethiopia, Kenya and Zambia [24, 26–28]. The scarcity of female health workers who are qualified to carry out skilled deliveries in our study area is apparent: in 2016, 33% of the 92 staff qualified to attend deliveries in CRR were female, in NBR-W, this was 40% of the 52 staff, and in URR it was 42% of the 77 staff.

Women’s reluctance to use donkey carts, the primary mode of transportation to health facilities in many of the study communities, influenced their decision to delay seeking care as much as the time to get to the health facility once transportation had been secured. Recognizing that this is an unacceptable mode of transportation for women in labor in this context is critical to informing appropriate interventions to address Delays 1 and 2.

The reported increase in the proportion of women accompanied and transported to delivery may represent a reduction in Delay 2 of getting to a health facility for delivery. Underlying causes of Delay 2 are well documented in the literature and correspond to our findings [8, 11, 13]. We found Delay 2 was exacerbated when labor commenced at night or during or after rains. A 2016 study of RBF communities in India found similar results [13], and a 2018 study of RBF communities in India found that transportation was less accessible at night and during holidays [11]. Barriers due to the cost of transportation also appeared in communities in India with RBF programs [8].

Qualitative data suggest that women receive prompt care upon arrival at health facilities – an important component of the third delay – particularly at the midline. Low utilization rates at the baseline evaluation may have contributed to this as they could have resulted in excess capacity leaving room to absorb increased uptake of services. However, although nurses were available in each of the three regions, the NBR-W and URR regions each had only one doctor, and no doctors were present in CRR. This shortage of senior staff may indicate insufficient capacity to provide more complex care for delivery complications, which was not assessed.

Previous studies have found that positive delivery outcomes influence women’s satisfaction with institutional delivery services, which may contribute to underreporting poor quality of care [29]. Reported improvements with quality of care may explain reduced delays in receiving skilled delivery care on arrival at the health facility. This may be a result of the national health policy encompassing attention to the availability of skilled birth attendants as well as recent improvements in quality of care. One important measure of the quality of care is the proportion of institutional deliveries carried out by skilled personnel, which increased substantially between baseline and midline (Table 4). It is important to recall that this was the indicator that was incentivized through the facility RBF intervention. However, health workers expressed concerns that insufficient supplies continue to detract from the quality of delivery services at health facilities and might impact the timeliness of care provision. Similarly, inadequate supplies at health facilities were described in RBF-receiving health facilities in India [11], and it is also a common issue in facilities not receiving RBF payments in Malawi and Ethiopia [25, 30].

### Implications for skilled delivery provision in The Gambia

Although no significant impact was found on rates of institutional or skilled delivery within the first 18 months, the RBF intervention increased both the proportion of women who reported having been referred to health facilities for delivery, as well as the proportion of women who reported having been accompanied and having been transported to the facility. Many communities reported having procured vehicles for transportation to the health facility, often using RBF subsidies to finance these vehicles. This may have contributed to the reduction of Delay 2 by 2016. The current low uptake of institutional delivery coupled with these data on intermediate outcomes of referral, accompaniment and transportation to the health facility for delivery, suggest that there might be an increase in facility deliveries in the near future.

Qualitative data indicate that, moving forward, focusing on Delays 1 and 2 while continuing to monitor availability of supplies and equipment may be an effective approach to increasing rates of institutional and skilled delivery. Though transportation procurement has seemingly reduced Delay 2, transportation difficulties continue to present significant challenges to skilled delivery uptake. Furthermore, although few delays were identified at the health facility, increasing the number of beds available for delivery, ensuring access to medications, and increasing the number of female health workers qualified to assist with deliveries is important to promote the provision of adequate care. The recruitment of skilled female health workers may help to increase demand for institutional deliveries and ensure timely receipt of quality care. This is supported by a 2015 study, which found that skilled female health workers significantly encouraged delivery at public health facilities in India [31].

This is a particularly critical issue in The Gambia at this time given the new role of CBCs, which is to conduct health promotion and advocacy activities rather than attend deliveries. A study in Peru noted a similar shift in the TBA role following an intervention that introduced a culturally appropriate delivery care model [32]. A 2007 ethnographic study of TBAs in The Gambia described them as the “gum that holds society together,” emphasizing social cohesion as an important function of this position [25]. Ten years later, and following the shift in “official” role, this function of community cohesion and social responsibility appears equally important and may underlie CBCs’ contentment with their new role. Understanding how best the CBCs might be supported to maintain their satisfaction and ensure an increase in skilled and facility delivery is critical to helping the country reach its targets relating to maternal mortality and population health promotion.

### Implications beyond The Gambia

The ‘three delays’ model remains a useful tool to understand when and how barriers to facility delivery occur. Using this model allows researchers and implementers to work together to explore reasons underlying delayed uptake of skilled delivery and target interventions to the specific local context. This model may even be useful beyond maternal mortality contexts, and has been adapted for use in global emergency health services [33].

This model also highlights useful lessons for the design of future RBF programs. Most RBF programs to date have focused on the provision of financial incentives within health facilities, with some also providing conditional cash transfers to women for attending health facilities for delivery. While the former might assist in reducing Delay 3 once women reach the health facility, and the latter might help women overcome cost barriers to attending the facility thus reducing Delay 2, additional delays, particularly surrounding women’s decision to seek care (Delay 1), may persist, limiting the overall project impact on uptake of institutional delivery as well as maternal and neonatal health outcomes. This underscores the importance of conducting SBCC activities in conjunction with the provision of financial incentives. Furthermore, both Delays 2 and 3 are often embedded in realities rooted at the househould and community levels. Recognizing this in The Gambia led to supplementing financial incentives to health facilities with financial incentives (and other supportive interventions) to communities to help reduce delays. As some of these underlying factors require collective community actions and changes in social norms, having a community focus helped in The Gambia and could be useful in other contexts as well, including for Delay 2 as many communities used these incentives to collectively purchase transportation for use by the entire community. Formative research on the three delays might help inform the design of RBF programs to appropriately target activities and incentives for maximum positive impact.

### Limitations

With only 19 health facilities included in the study, the ability to detect statistically significant change due to the intervention within this 18-month period is limited. This might help explain why no significant change was found in the uptake of institutional or skilled delivery. The significant improvements in intermediate outcomes, coupled with the qualitative findings suggest that a shift towards institutional and skilled delivery might be taking place.

While the sample was restricted to women who had delivered in the 450 days prior to survey administration, some of the women interviewed at midline would not have been exposed to the full range of project interventions throughout their entire pregnancy, which may lead us to under-estimate the overall effect size of the intervention.

A risk of desirability bias exists if participants were under the impression that giving a favorable evaluation of the project might lead to additional funding and/or activities. The research team was trained to clearly explain the independence of the evaluation from the project itself so as to militate against this risk.

## Conclusions

This research suggests that improvements in some of the ‘three delays’ could be detected between the two survey rounds, and the model points to opportunities to strengthen implementation to address other causes underlying these delays in an effort to increase skilled delivery. The ‘three delays’ model continues to be a useful framework for understanding where bottlenecks lie in increasing skilled deliveries and for informing efforts to address these moving forward.

## Data Availability

The data collection instruments used during the current study are available from World Bank’s RBF for Health Microdata Repository on reasonable request: http://microdata.worldbank.org/index.php/home. The baseline data are publicly available and access to the midline data can be requested through this link.

## Abbreviations

ANC: Antenatal care
CBC: Community birth companion
CCT: Conditional cash transfer
CRR: Central River Region
DEFF: Design effect
FGD: Focus group discussion
MCNHRP: Maternal and child nutrition and health results project
MRC: Medical Research Council
NBR-W: North Bank Region-West
NGO: Non-Governmental Organization
RBF: Results based financing
RHD: Regional Health Directorate
SBCC: Social and behavioural change communication
TBA: Traditional Birth Attendant
URR: Upper River Region
VDC: Village development committee
VSG: Village support group
